# Magnitude of potentially inappropriate prescribing in Germany among older patients with generalized anxiety disorder

**DOI:** 10.1186/1471-2318-9-31

**Published:** 2009-07-27

**Authors:** Ariel Berger, Marko Mychaskiw, Ellen Dukes, John Edelsberg, Gerry Oster

**Affiliations:** 1 Policy Analysis, Inc, Brookline, USA; 2 Worldwide Medical and Outcomes Research, Pfizer, Inc, New York City, USA; 3 Global Outcomes Research, Pfizer, Inc New York City, USA

## Abstract

**Background:**

Several medications commonly used to treat generalized anxiety disorder (GAD) have been designated "potentially inappropriate" for use in patients aged ≥65 years because their risks may outweigh their potential benefits. The actual extent of use of these agents in clinical practice is unknown, however.

**Methods:**

Using a database with information from encounters with general practitioners (GP) in Germany, we identified all patients, aged ≥65 years, with any GP office visits or dispensed prescriptions with a diagnosis of GAD (ICD-10 diagnosis code F41.1) between 10/1/2003 and 9/30/2004 ("GAD patients"). Among GAD-related medications (including benzodiazepines, tricyclic antidepressants [TCAs], selective serotonin reuptake inhibitors, venlafaxine, hydroxyzine, buspirone, pregabalin, and trifluoperazine), long-acting benzodiazepines, selected short-acting benzodiazepines at relatively high dosages, selected TCAs, and hydroxyzine were designated "potentially inappropriate" for use in patients aged ≥ 65 years, based on published criteria.

**Results:**

A total of 975 elderly patients with GAD were identified. Mean age was 75 years, and 72% were women; 29% had diagnoses of comorbid depression. Forty percent of study subjects received potentially inappropriate agents – most commonly, bromazepam (10% of all subjects), diazepam (9%), doxepin (7%), amitriptyline (5%), and lorazepam (5%). Twenty-three percent of study subjects received long-acting benzodiazepines, 10% received short-acting benzodiazepines at relatively high doses, and 12% received TCAs designated as potentially inappropriate.

**Conclusion:**

GPs in Germany often prescribe medications that have been designated as potentially inappropriate to their elderly patients with GAD – especially those with comorbid depressive disorders. Further research is needed to ascertain whether there are specific subgoups of elderly patients with GAD for whom the benefits of these medications outweigh their risks.

## Background

Generalized anxiety disorder (GAD) is a chronic condition that is characterized by persistent worry or anxiety that occurs more days than not over a period of at least six months [1]. The condition is frequently difficult to diagnose because of the variety of clinical presentations and the common occurrence of comorbid medical or other psychiatric conditions. Lifetime prevalence has been estimated to be between 4% and 6% [2]; the disease is more common among women than men. GAD is the most common anxiety disorder among patients presenting to primary care physicians [3,4].

Several different types of medications are often used to treat GAD – specifically, benzodiazepines (e.g., flurazepam, diazepam, chlordiazepoxide), buspirone, tricyclic antidepressants (TCAs) (e.g., amitriptyline, imipramine, doxepin, opipramol), selective serotonin reuptake inhibitors (SSRIs) (e.g., paroxetine. escitalopram), and venlafaxine (a selective serotonin and norepinephrine reuptake inhibitor) [5-7]. Among these available therapies, benzodiazepines have long been the mainstay of pharmacologic treatment for GAD. While effective, benzodiazepines are associated with excessive sedation and motor impairment [8]; their long-term use is also associated with a risk of physical dependence as well as withdrawal when therapy is discontinued [6]. In one study comparing 4554 persons prescribed benzodiazepines with 13,662 persons receiving other (i.e., non-benzodiazepine) medications who were matched on age, sex, and calendar month in which therapy was initiated, Oster and colleagues found that patients in the former group had a 15% higher risk of an accident-related medical event; those who filled three or more prescriptions for benzodiazepines had a 30% higher risk compared with those who filled only one such prescription [9].

An expert panel convened by Beers in 1991 developed explicit criteria for identifying medication use among nursing home residents that was potentially inappropriate [10]. Recognizing that these criteria were developed specifically for a nursing home population, Beers convened another expert panel in 1997 to develop criteria applicable to the entire population of older persons (≥65 years); the resulting criteria designated some of the drugs used to treat GAD (benzodiazepines, amitriptyline, doxepin) as potentially inappropriate for use in persons aged ≥ 65 years [11]. The panel compiled its list of potentially inappropriate medications without regard to diagnosis or place of residence, and sought to include only those agents whose ". . . potential for adverse outcomes is greater than the potential for benefit" [11].

While well-known and extensively cited, the Beers' criteria have been criticized as not providing a sufficient basis for identifying inappropriate prescribing, as they are not indication-specific [12]. A subsequent expert panel convened by Zhan et al. classified 33 medications on the Beers' list alternatively as always to be avoided, rarely appropriate, and appropriate for some indications [13]. Among drugs that are sometimes used to treat GAD, flurazepam was designated as "always to be avoided"; chlordiazepoxide and diazepam were designated as "rarely appropriate"; and amitriptyline and doxepin, "appropriate for some indications".

In their update of the Beers' criteria, Fick et al. designated flurazepam, amitriptyline, chlordiazepoxide, doxepin, and anything other than low doses of short-acting benzodiazepines (e.g., >3 mg lorazepam) as potentially inappropriate for use in older patients; adverse outcomes for all such medications were deemed by the authors to be of high (versus low) severity [14].

Despite their limitations, the 1997 Beers' criteria have been widely used by researchers to identify potential medication risks [13,15-19]. An epidemiologic study of noninstitutionalized persons who participated in the 1987 US National Medical Expenditure Survey reported that 23.5% of those aged ≥65 years received at least one of the 20 medications on the Beers' list [20]. Zhan et al. applied their revised list to persons participating in the 1996 US Medical Expenditure Panel Survey and reported that 21.3% received drugs that were potentially – albeit not necessarily – inappropriate, 2.6% received medications that should always be avoided, and 9.1% received drugs that were rarely appropriate; 3.4% of those aged >65 years had received amitriptyline [13].

Recently, an examination of 2707 older home-care patients from eight countries across Europe found that 19.8% received at least one medication designated as potentially inappropriate; in multivariate analysis, use of anxiolytics was associated with a twofold increase in the likelihood of receiving potentially inappropriate medications [21]. Some of the most commonly used, potentially inappropriate medications in this study were benzodiazepines (diazepam [3.1% of patients received such therapy] and chlordiazepoxide [0.6%]) and tricyclic antidepressants (amitriptyline [1.4%]) – agents often used for the treatment of GAD.

Although older adults with GAD would appear to often receive potentially inappropriate medications, the actual extent of such use in clinical practice is unknown. Moreover, the generalizability of earlier findings – based largely on US data – to other countries is unknown. In this study, we examine the magnitude of exposure of patients aged ≥65 years with GAD to potentially inappropriate medications in Germany, a large European country in which observed patterns of treatment of GAD may possibly be reflective of those throughout Europe.

## Methods

Data were obtained from the IMS MediPlus – Disease Analyzer database; a detailed description of the database and its research capabilities may be found elsewhere [22]. The database is longitudinal in nature, and provides patient-level information on consultations, diagnoses, and treatments from over 900 GP practices throughout Germany, comprising more than 4.2 million patient records and 75 million prescriptions over a 10-year period. The database is compiled by sampling practices throughout Germany, and is designed to be representative of the general population in Germany. All patient identifiers in the database are fully encrypted.

Information in the IMS MediPlus – Disease Analyzer database includes date of service, diagnoses (in ICD-10 format), actions taken (e.g., referrals to other providers [i.e., specialists], dispensing of sick notes [physician-excused absences from work]), and medications dispensed, including the dispensing date, the quantity dispensed, the number of days of therapy supplied, and the associated diagnosis (available for about 60% of all prescription records). Selected demographic information is also available, including patient age and gender. All patient-level data can be arrayed chronologically to provide a detailed, longitudinal profile of all medical and pharmacy services rendered by participating GPs. Because this study was retrospective in nature, used completely anonymized data, and did not involve patient contact, institutional review board (IRB) approval was not sought. The database for this study encompassed the period, October 1, 2003 through September 30, 2004 ("study period").

The study sample consisted of all patients with any GP office visits or dispensed prescriptions with a diagnosis of GAD (ICD-10 diagnosis code F41.1) during the study period. Persons aged <65 years as of their first-noted encounter for GAD were excluded from the study sample. All GP encounters were then compiled for all subjects over the one-year period of study.

The prevalence of a number of medically attended comorbidities (i.e., noted by GPs during office or clinic visits) was examined for patients in the study sample, including: (1) neoplasms; (2) anemia and other blood antibody disorders; (3) diabetes; (4) circulatory system disorders; (5) respiratory system disorders; (6) eye, nose, and throat disorders; (7) digestive system disorders; (8) painful neuropathic disorders (e.g., diabetic peripheral neuropathy, post-herpetic neuralgia, causalgia, neuropathic back pain); (9) musculoskeletal system disorders; (10) symptom, signs, ill-defined conditions; (11) somatoform disorders; (12) neurasthenia; (13) substance use disorders; and (14) sleep disorders. Patients were deemed to have these conditions if they had *any *encounters during the study period with the corresponding diagnosis code(s).

The numbers of patients receiving various medications often used to treat GAD ("GAD-related medications") were tabulated. Medications (and corresponding dosages, if relevant [see below]) were deemed "potentially inappropriate" based on their inclusion in Beers' 1997 criteria and/or in subsequent updates to these criteria [11,13,14]. Medication regimens designated as potentially inappropriate were as follows: (1) alprazolam (>2 mg daily); (2) amitriptyline; (3) chlorazepate; (4) chlordiazepoxide; (5) diazepam; (6) doxepin; (7) flurazepam; (8) halazepam; (9) lorazepam (>3 mg daily); (10) oxazepam (>60 mg daily); (11) temazepam (>15 mg daily); (12) triazolam (>0.25 mg daily); and (13) zolpidem (>5 mg daily) [Table 1]. Daily dose was calculated using information in the database; in instances where such information was missing, daily dose was assumed equivalent to the modal value from all other prescriptions for the same product with non-missing values.

**Table 1:** Medications for treatment of generalized anxiety disorder

**Medication Type**	**Comment**
Benzodiazepines	
Short-acting	
Alprazolam	>2 mg/d deemed potentially inappropriate
Bromazepam	
Lorazepam	>3 mg/d deemed potentially inappropriate
Lormetazepam	
Nitrazepam	
Oxazepam	>60 mg/d deemed potentially inappropriate
Temazepam	>15 mg/d deemed potentially inappropriate
Tetrazepam	
Triazolam	>0.25 mg/d deemed potentially inappropriate
Zolpidem	>5 mg/d deemed potentially inappropriate
Long-acting	
Chlordiazepoxide	All long-acting benzodiazepines deemed potentially inappropriate
Chlorazepate	All long-acting benzodiazepines deemed potentially inappropriate
Clobazam	All long-acting benzodiazepines deemed potentially inappropriate
Clonazepam	All long-acting benzodiazepines deemed potentially inappropriate
Diazepam	All long-acting benzodiazepines deemed potentially inappropriate
Flunitrazepam	All long-acting benzodiazepines deemed potentially inappropriate
Flurazepam	All long-acting benzodiazepines deemed potentially inappropriate
Halazepam	All long-acting benzodiazepines deemed potentially inappropriate
Medazepam	All long-acting benzodiazepines deemed potentially inappropriate
Nordazepam	All long-acting benzodiazepines deemed potentially inappropriate
Prezepam	All long-acting benzodiazepines deemed potentially inappropriate
Tricyclic antidepressants	
Amitriptyline	Deemed potentially inappropriate
Amitriptylinoxide	
Clomipramine	
Dibenzepin	
Desipramine	
Doxepin	Deemed potentially inappropriate
Imipramine	
Maprotiline	
Nortiptyline	
Opipramol	
Trimipramine	
Selective serotonin reuptake inhibitor	
Escitalopram	
Citalopram	
Fluoxetine	
Fluvoxamine	
Paroxetine	
Sertraline	
Venlafaxine	
Hydroxyzine	Deemed potentially inappropriate
Buspirone	
Pregabalin	
Trifluoperazine	

Medication receipt was ascertained for the overall study sample, as well as within strata defined on the basis of age, sex, and selected comorbidities (e.g., depression, neoplasms, respiratory disorders). The statistical significance of differences within strata was ascertained using chi-square tests and Fisher's exact tests, as appropriate. All analyses were conducted using PC-SAS^® ^v.9.1 [23].

## Results

The study sample consisted of 975 patients, aged ≥65 years, with diagnoses of GAD; mean (± SD) age was 75.0 (± 7.3) years, and 71.6% were women [Table 2]. Twenty-four percent of study subjects had at least one encounter during the study period at which a diagnosis of another anxiety disorder was noted; 29.2% had at least one encounter resulting in a diagnosis of depression. The prevalence of other medically attended comorbidities was high, including circulatory disorders (87.5%), digestive disorders (56.0%), respiratory disorders (42.5%), musculoskeletal disorders (69.5%), painful neuropathies (28.8%), and sleep disorders (24.7%). Ninety-eight percent of patients had at least one comorbidity, and 87.0% had three or more.

**Table 2:** Demographic and clinical characteristics of study subjects (N = 975)*

**Characteristic**	
Age, years	
65–74	507 (52.0)
75–84	365 (37.4)
≥85	103 (10.6)
Mean (SD)	75.0 (7.3)
Females	698 (71.6)
Males	277 (28.4)
Comorbid conditions	
Anxiety disorders	
Panic disorder	17 (1.7)
OCD	2 (0.2)
PTSD	0 (0.0)
Phobias	
Social phobia	0 (0.0)
Agoraphobia	0 (0.0)
All other phobias	10 (1.0)
Any phobia	10 (1.0)
Other anxiety disorders	215 (22.1)
Any anxiety disorder (other than GAD)	237 (24.3)
Depression disorders	
Dysthymic disorder	10 (1.0)
Adjustment disorder with depression	4 (0.4)
Bipolar depression	1 (0.1)
MDD	36 (3.7)
Unspecified depression	256 (26.3)
Any depression	285 (29.2)
Neoplasms	158 (16.2)
Anemia and other blood/antibody disorders	136 (13.9)
Diabetes	269 (27.6)
Circulatory system disorders	853 (87.5)
Respiratory system disorders	414 (42.5)
Eyes, nose, and throat	262 (26.9)
Digestive system disorders	546 (56.0)
Painful neuropathic disorders	281 (28.8)
Musculoskeletal system disorders	679 (69.6)
Symptoms, signs, ill-defined conditions	610 (62.6)
Somatoform disorders	130 (13.3)
Neurasthenia	39 (4.0)
Substance use disorders	30 (3.1)
Sleep disorders	241 (24.7)
Number of comorbidities	
0	17 (1.7)
1	44 (4.5)
2	66 (6.8)
≥3	848 (87.0)

*Unless otherwise indicated, all values represent number (%)

GAD: Generalized anxiety disorder; OCD: Obsessive-compulsive disorder; PTSD: Post-traumatic stress disorder; MDD: Major depressive disorder

A total of 607 study subjects (62.3%) had received one or more GAD-related medications (both those deemed potentially inappropriate and all others) from their GPs – most commonly, benzodiazepines (43.7%), including both short-acting (24.8%) and long-acting (23.1%) formulations, and TCAs (25.6%) [Table 3]. Nine percent of patients received SSRIs, and 0.9% received venlafaxine.

**Table 3:** Numbers of study subjects receiving potentially inappropriate medications for treatment of GAD*

**Medication**	**Potentially Inappropriate**	**Possibly Appropriate**	**Total**
Benzodiazepines			
Short-acting			
Alprazolam^1^	0 (0.0)	29 (3.0)	29 (3.0)
Lorazepam^2^	50 (5.1)	21 (2.2)	71 (7.3)
Lormetazepam	14 (1.4)	0 (0.0)	14 (1.4)
Oxazepam^3^	0 (0.0)	88 (9.0)	88 (9.0)
Temazepam^4^	8 (0.8)	0 (0.0)	8 (0.8)
Tetrazepam	0 (0.0)	29 (3.0)	29 (3.0)
Triazolam^5^	0 (0.0)	1 (0.1)	1 (0.1)
Zolpidem^6^	32 (3.3)	3 (0.3)	34 (3.5)
Any of above	97 (9.9)	162 (16.6)	242 (24.8)
Long-acting**			
Chlordiazepoxide	6 (0.6)	---	6 (0.6)
Chlorazepate	11 (1.1)	---	11 (1.1)
Clobazam	2 (0.2)	---	2 (0.2)
Clonazepam	3 (0.3)	---	3 (0.3)
Diazepam	91 (9.3)	---	91 (9.3)
Flunitrazepam	7 (0.7)	---	7 (0.7)
Flurazepam	6 (0.6)	---	6 (0.6)
Halazepam	0 (0.0)	---	0 (0.0)
Medazepam	5 (0.5)	---	5 (0.5)
Nordazepam	2 (0.2)	---	2 (0.2)
Prezepam	0 (0.0)	---	0 (0.0)
Bromazepam	93 (9.5)	---	93 (9.5)
Nitrazepam	14 (1.4)	---	14 (1.4)
Midazolam	0 (0.0)	---	0 (0.0)
Any of above	225 (23.1)	---	225 (23.1)
Any of above	298 (30.6)	162 (16.6)	426 (43.7)
Tricyclic antidepressants			
Amitriptyline	52 (5.3)	---	52 (5.3)
Amitriptylinoxide	0 (0.0)	3 (0.3)	3 (0.3)
Clomipramine	0 (0.0)	4 (0.4)	4 (0.4)
Dibenzepin	0 (0.0)	3 (0.3)	3 (0.3)
Desipramine	0 (0.0)	0 (0.0)	0 (0.0)
Doxepin	70 (7.2)	---	70 (7.2)
Imipramine	0 (0.0)	0 (0.0)	0 (0.0)
Maprotiline	0 (0.0)	4 (0.4)	4 (0.4)
Nortriptyline	0 (0.0)	0 (0.0)	0 (0.0)
Opipramol	0 (0.0)	105 (10.8)	105 (10.8)
Trimipramine	0 (0.0)	29 (3.0)	29 (3.0)
Any of above	119 (12.2)	142 (14.6)	250 (25.6)
Selective serotonin reuptake inhibitors			
Escitalopram	0 (0.0)	7 (0.7)	7 (0.7)
Citalopram	0 (0.0)	40 (4.1)	40 (4.1)
Fluoxetine	11 (1.1)	0 (0.0)	11 (1.1)
Fluvoxamine	0 (0.0)	3 (0.3)	3 (0.3)
Paroxetine	0 (0.0)	16 (1.6)	16 (1.6)
Sertraline	0 (0.0)	13 (1.3)	13 (1.3)
Any of above	11 (1.1)	77 (7.9)	86 (8.8)
Venlafaxine	0 (0.0)	9 (0.9)	9 (0.9)
Hydroxyzine	4 (0.4)	---	4 (0.4)
Buspirone	0 (0.0)	5 (0.5)	5 (0.5)
Pregabalin	0 (0.0)	0 (0.0)	0 (0.0)
Trifluoperazine	0 (0.0)	0 (0.0)	0 (0.0)
Any of above	392 (40.2)	345 (35.4)	607 (62.3)

*All values are number of patients (%); dose calculated using all values found in database

**Any use of long-acting benzodiazepines deemed potentially inappropriate

^1^Dose >2 mg/d deemed potentially inappropriate

^2^Dose >3 mg/d deemed potentially inappropriate

^3^Dose >60 mg/d deemed potentially inappropriate

^4^Dose >15 mg/d deemed potentially inappropriate

^5^Dose > 0.25 mg/d deemed potentially inappropriate

^6^Dose >5 mg/d deemed potentially inappropriate

GAD: Generalized anxiety disorder

About forty percent of study subjects – or two-thirds of patients (64.6%) with evidence of receipt of GAD-related medications – were dispensed at least one therapy considered potentially inappropriate for use in the elderly. Ten percent of patients received short-acting benzodiazepines at dosages that rendered their use potentially inappropriate, 23.1% received long-acting benzodiazepines, and 12.2% received amitriptyline or doxepin (primarily doxepin).

Receipt of potentially inappropriate therapy did not differ by age (39.1% for patients aged 65–74 years vs 41.5% for patients aged ≥75 years; p = 0.45) or gender (41.1% of men vs 37.9% of women; p = 0.36). Patients with comorbid depression were more likely to receive potentially inappropriate therapy (51.6% vs 35.5% for those without comorbid depression; p < 0.01), as were those with digestive system disorders (43.0% vs 36.6% for those without these disorders; p = 0.04), and those with sleep disorders (62.2% vs 33.0% for those without these disorders; p < 0.01); receipt of these therapies did not differ significantly for patients with versus without any of the other selected comorbidities.

## Discussion

GAD can be difficult to treat, and several different medications – including benzodiazepines, buspirone, TCAs, and SSRIs – are recommended for use in these patients [5-7]. Although these therapies are often of benefit, they also can confer significant risks in older adults. Because of these risks, some of these drugs have been designated potentially inappropriate for use in persons aged 65 years or older.

Using the 1997 Beers criteria [11] and subsequent updates [13,14], we found that four out of every 10 patients – and two out of every three of those who received any GAD-related therapy – received medications that have been designated as potentially inappropriate for use among persons aged 65 years and older. Notably, use of these medications did not differ between the "young" old versus the "old" old (i.e., aged 65–74 years vs ≥ 75 years).

Benzodiazepines have long been the mainstay of pharmacologic treatment for GAD, and they were the medication most commonly dispensed among subjects in our study: 44% of patients in our study received these agents. Paradoxically, they also comprise the majority of agents deemed potentially inappropriate for use in the elderly, due in part to an increased risk of falls, hip fractures, drug-induced disorders of cognition, and motor vehicle accidents [9,24-27]. We note that GPs dispensed short-acting benzodiazepines at daily doses deemed potentially inappropriate to 10% of study subjects; they also dispensed long-acting benzodiazepines – which are deemed potentially inappropriate in older patients regardless of daily dose – to 31% of patients. About one in 10 patients in our study received TCAs deemed potentially inappropriate – either amitriptyline or doxepin – despite the fact that several other TCAs (e.g., nortriptyline) are available with similar efficacy that are better tolerated by older patients. We are unaware of any other study of potentially inappropriate prescribing in patients (of any age) with GAD. Compared with rates of potentially inappropriate prescribing reported among the elderly in general [13,16-21,28], the rates we report are considerably higher. This finding is consistent, however, with the fact that many of the medications that have been designated by some experts as potentially inappropriate in the elderly are also commonly recommended by other experts to treat GAD.

As people age, their ability to metabolize drugs decreases, and receptor sensitivity to the effects of pharmacotherapy changes [29,30]. Unfortunately, older adults are often underrepresented in randomized controlled trials [15], an important source of information for prescribers. Consequently, physicians may sometimes prescribe therapies on presumptions of efficacy and safety based on clinical trial results that are not necessarily generalizable to persons of advanced age. This may explain why we found that so many patients with GAD received medications that were potentially inappropriate. Inappropriate prescribing also may result from the use of inappropriate doses of otherwise age-appropriate medications and/or not prescribing medications that may be of benefit (e.g., not prescribing GAD-related therapies for patients with this condition) [31]. Accordingly, our findings probably underestimate the magnitude of potentially inappropriate prescribing in Germany among older patients with GAD. Further study is needed to ascertain the extent to which these other aspects of potentially inappropriate prescribing are evident in this patient population.

The clinical implication of our findings appears to be that benzodiazepines, amitriptyline, and doxepin are being overprescribed in Germany among elderly patients with GAD, especially among those with comorbid depression and/or sleep disorders. A major limitation of our study, however, is that we cannot assess the actual extent to which prescribing was truly clinically inappropriate. The treatment of GAD in the elderly is clinically complex and presents many challenges. Since we did not employ a "therapeutic timeline" as part of our research design, patients could have received potentially inappropriate agents either as initial treatment or following failure of safer, more appropriate agents. To the extent that the latter occurred, providers may have known about the risks associated with these medications and nonetheless used them only after a careful balancing of these risks against their potential benefits in patients with severe symptoms [29]. Further study is needed to clarify the extent of truly clinically inappropriate prescribing.

A second limitation of our study is that the list of potentially inappropriate agents may be too rigid. For example, while Beers classified amitriptyline as potentially inappropriate, Zhan et al. [13] deemed the use of low doses of this agent as appropriate in older adults in some instances, such as for the treatment of neuropathic pain. We note, however, that the Beers criteria are not disease-specific; these agents are deemed potentially inappropriate based exclusively on age ≥65 years. Furthermore, the panel convened by Zhan et al. concluded that drugs such as amitriptyline "are often misused in clinical practice" [13].

Finally, we note that the database was limited to information from encounters with GPs. Patients who were seen for the treatment of GAD by psychiatrists may not have been included in our study (unless they also received a diagnosis of GAD from their GPs). Moreover, all information on prescription drugs is limited to those that are dispensed by GPs. The generalizability of our findings to all older patients with GAD is therefore unknown.

## Conclusion

GPs in Germany often prescribe medications that have been designated as potentially inappropriate to their elderly patients with GAD – especially those with comorbid depressive disorders. Further research is needed to ascertain whether there are specific subgoups of elderly patients with GAD for whom the benefits of these medications outweigh their risks.

## Competing interests

Funding for this research was provided by Pfizer Inc., New York, NY. Marko Mychaskiw, R.Ph., Ph.D., and Ellen Dukes, Ph.D., are employees of Pfizer Inc. as well as co-authors of this manuscript; they were involved with the design of the study, data analysis and interpretation, manuscript preparation, and publication decisions. Ariel Berger, M.P.H., John Edelsberg, M.D., M.P.H., and Gerry Oster, Ph.D., are employees of Policy Analysis, Inc., who were paid consultants to Pfizer in consultation with the development of this manuscript.

## Authors' contributions

AB, MM, ED, JE, and GO all contributed to the conceptualization and design of study, data interpretation, and manuscript preparation. In addition, AB, JE, and GO undertook all statistical analyses of the data. All authors read and approved the final manuscript.

## Pre-publication history

The pre-publication history for this paper can be accessed here:



## References

[B1] American Psychiatric Association (2000). Diagnostic and Statistical Manual of Mental Disorders, Fourth Edition, Text Revision.

[B2] Kessler R, Berglund P, Demler O (2005). Lifetime prevalence and age of onset distributions of DSM-IV disorders in the National Comorbidity Survey Replication. Arch Gen Psychiatry.

[B3] Ormel J, VonKorff M, Ustun T (1994). Common mental disorders and disability across cultures: Results from the WHO collaborative study on psychological problems in general health care. JAMA.

[B4] Wittchen HU, Kessler RC, Beesdo K (2002). Generalized anxiety and depression in primary care: Prevalence, recognition, and management. J Clin Psychiatry.

[B5] Baldwin DS, Anderson IM, Nutt DJ (2005). Evidence-based guidelines for the pharmacological treatment of anxiety disorders: Recommendations from the British Association for Psychopharmacology. J Psychopharmacol.

[B6] Gorman JM (2003). Treating generalized anxiety disorder. J Clin Psychiatry.

[B7] Rickels K, Schweizer E (1998). The spectrum of generalised anxiety in clinical practice: The role of short-term, intermittent treatment. Br J Psych.

[B8] Shader RI, Greenblatt DJ (1993). Use of benzodiazepines in anxiety disorders. N Engl J Med.

[B9] Oster G, Huse DM, Adams SF (1990). Benzodiazepine tranquilizers and the risk of accidental injury. Am J Pub Health.

[B10] Beers MH, Ouslander JG, Rollinger I (1991). Explicit criteria for determining inappropriate medication use in nursing home residents. Arch Intern Med.

[B11] Beers MH (1997). Explicit criteria for determining potentially inappropriate medication use by the elderly: An update. Arch Intern Med.

[B12] Anderson GM, Beers MH, Kerluke K (1997). Auditing prescription practice using explicit criteria and computerized drug benefit claims data. J Eval Clin Pract.

[B13] Field TS, Gurwitz JH, Glynn RJ (1999). The renal effects of nonsteroidal anti-inflammatory drugs in older people: Findings from the Established Populations for Epidemiologic Studies of the Elderly. J Am Geriatr Soc.

[B14] Fick DM, Cooper JW, Wade WE (2003). Updating the Beers Criteria for potentially inappropriate medication use in older adults: Results of a US consensus panel of experts. Arch Intern Med.

[B15] Gurwitz JH, Avorn J (1991). The ambiguous relation between aging and adverse drug reactions. Ann Intern Med.

[B16] Spore DL, Mor V, Larrat P (1997). Inappropriate drug prescriptions for elderly residents of board and care facilities. Am J Public Health.

[B17] Aparasu RR, Mort JR (2000). Inappropriate prescribing for the elderly: Beers criteria-based review. Ann Pharmacother.

[B18] Liu GG, Christensen DB (2002). The continuing challenge of inappropriate prescribing in the elderly: An update of the evidence. J Am Pharm Assoc.

[B19] Caterino JM (2003). Administration of inappropriate medications to elderly emergency department patients: Results of a national survey. Acad Emerg Med.

[B20] Willcox SM, Himmelstein DU, Woolhandler S (1994). Inappropriate drug prescribing for the community-dwelling elderly. JAMA.

[B21] Fialova D, Topinkova E, Gambassi G (2005). Potentially inappropriate medication use among elderly home care patients in Europe. JAMA.

[B22] Dietlein G, Schroder-Bernhardi D (2002). Use of the Mediplus^® ^patient database in healthcare research. Int J Clin Pharm Ther.

[B23] SAS® Proprietary Software, Release 84.

[B24] Cumming RG, LeCouteur DG (2003). Benzodiazepines and risk of hip fractures in older people: A review of the evidence. CNS Drugs.

[B25] Petrovic M, Mariman A, Warie H (2003). Is there a rationale for prescription of benzodiazepines in the elderly? Review of the literature. Acta Clin Belg.

[B26] Gray SL, Lai KV, Larson EB (1999). Drug-induced cognition disorders in the elderly: Incidence, prevention and management. Drug Saf.

[B27] Thomas RE (1998). Benzodiazepine use and motor vehicle accidents. Systematic review of reported association. Can Fam Physician.

[B28] Gurwitz JH (1994). Suboptimal medication use in the elderly. The tip of the iceberg. JAMA.

[B29] (2002). AGS Panel on Persistent Pain in Older Persons. The management of persistent pain in older persons. J Am Geriatr Soc.

[B30] Knight EL, Avorn J (2001). Quality indicators for appropriate medication use in vulnerable elders. Ann Intern Med.

[B31] Hamilton HJ, Gallagher PF, O'Mahony D (2009). Inappropriate prescribing and adverse drug events in older people. BMC Geriatrics.

